# The Potential Role of Vitamin D Enhanced Foods in Improving Vitamin D Status

**DOI:** 10.3390/nu3121023

**Published:** 2011-12-16

**Authors:** Louise O’Mahony, Magdalena Stepien, Michael J. Gibney, Anne P. Nugent, Lorraine Brennan

**Affiliations:** 1 UCD Institute of Food and Health, University College Dublin, Belfield, Dublin 4, Ireland, Email: louise.omahony@ucd.ie (L.O.M.); magdalena.stepien@ucd.ie (M.S.); mike.gibney@ucd.ie (M.J.G.); anne.nugent@ucd.ie (A.P.N.); 2 UCD Conway Institute, University College Dublin, Belfield, Dublin 4, Ireland

**Keywords:** vitamin D, 25(OH)D, enhanced foods, fortification

## Abstract

Low vitamin D intake and status have been reported worldwide and many studies have suggested that this low status may be involved in the development of several chronic diseases. There are a limited number of natural dietary sources of vitamin D leading to a real need for alternatives to improve dietary intake. Enhancement of foods with vitamin D is a possible mode for ensuring increased consumption and thus improved vitamin D status. The present review examines studies investigating effects of vitamin D enhanced foods in humans and the feasibility of the approach is discussed.

## 1. Introduction

Vitamin D generally refers to two fat soluble prohormones, ergocalciferol (vitamin D_2_) and cholecalciferol (vitamin D_3_). Vitamin D_2_ is produced by certain invertebrates and fungi whilst vitamin D_3_ is produced in the skin of vertebrates, both following exposure to ultraviolet (UV) radiation. Ingested vitamin D_2_ and endogenously produced D_3_ are converted to the biologically active form, 1,25-dihydroxyvitamin D [1,25(OH)2D] (calcitriol) in the human body. 

Vitamin D is responsible for a wide range of functions in the body and these known roles are expanding as research in this field accumulates. The discovery in 1969 of the nuclear vitamin D receptor (VDR) for 1,25(OH)_2_D stimulated a vast amount of research that to date has collectively described the presence of the VDR in at least 38 human tissues and organs [1]. The VDR was first described in the bone, kidney and gastrointestinal tract but since has been identified in a diverse range of other tissues including those in the brain, breast, colon and prostate. The VDR, a phosphoprotein, facilitates the various biological functions of calcitriol by binding to the active hormone with high affinity and regulating the expression of genes via zinc finger-mediated DNA binding and protein-protein interactions [2]. 

The well established functions of vitamin D relate to calcium absorption and homeostasis, with the role for vitamin D in bone mineralization and bone health extensively documented and long recognised, particularly in relation to rickets and osteomalacia [3,4]. Vitamin D, in tandem with calcium, may also be involved in the prevention of osteoporosis, fracture incidence and falls, particularly in older populations [5,6]. However, vitamin D is no longer regarded as just a calcaemic hormone as a result of the widespread distribution of the VDR and the fact that the consequences of insufficiency have been shown to extend beyond the above classic effects. Though the evidence is less robust, vitamin D deficiency is now implicated in a host of other diseases including psoriasis, multiple sclerosis, inflammatory bowel disease, type 1 and 2 diabetes, hypertension, cardiovascular disease, the metabolic syndrome and various cancers [7,8]. A thorough examination of all these functions is beyond the scope of the present review and the reader is referred to a number of good quality reviews [7,9]. 

In parallel with the growing evidence for widespread action for vitamin D there is robust data demonstrating that an increased vitamin D status is linked to significant benefits in terms of morbidity and mortality risk reduction. A recent review demonstrated that an estimated 20% reduction of global vitamin D-sensitive disease mortality and up to 17.6% of all-cause mortality could be achieved by doubling vitamin D status [10]. In Nordic populations improvement of vitamin D status may decrease the risk of mortality from a number of common diseases including cancer and cardiovascular disease between 19 and 24%, depending on the country [10]. For Canada, which shares a similar northerly latitude, reductions of 16.1% were estimated [11]. These findings corroborate those of a number of other studies that have demonstrated that mortality is greatest amongst individuals with the lowest vitamin D status [12,13]. 

Taking into account the multiple functions of vitamin D and the disease risk-reduction potential, it has become evident that maintaining an adequate status is important. Vitamin D status is assessed by measuring the circulating form of the vitamin (25(OH)D) in serum. There is much debate in the literature as to which cut-offs to use for defining vitamin D deficiency. However, recent research has lead investigators to increase the cut-off for deficiency to 50 nmol/L (from 25 nmol/L). Concomitant with this, serum concentrations of 75 nmol/L or higher have been established as adequate levels, with values in between indicating insufficiency [14,15,16].

Estimates of global vitamin D status indicate widespread inadequacy, with severe hypovitaminosis D most common in the Middle East and South Asia [15]. Even within Europe, large variance in serum concentrations exist between countries [17]. Though individuals living at higher latitudes commonly exhibit low 25(OH)D serum concentrations due to reduced UVB radiation, especially during winter months [18,19,20], high levels of inadequacy have also been found in Southern Europe, particularly among older populations [21,22]. In the UK, estimates suggest that only 18% and 24.1% of women and men, respectively, have adequate 25(OH)D concentrations [19]. Higher 25(OH)D levels have been reported in Canada, where 35% of the population have levels above 75 nmol/L [23]. In the US, 58.4% and 27% of adults have serum 25(OH)D concentrations >50 nmol/L and >75 nmol/L, respectively [24,25]. 

Stemming from the reports of low levels of circulating 25(OH)D, certain countries recommend an exposure of 10-15 min daily of sunshine [26,27]. However, due to geographical, cultural and behavioural differences between populations, increased UVB radiation exposure is not sufficient to maintain healthy vitamin D levels, especially during winter. Therefore, optimal dietary vitamin D intake is crucial to maintain adequate vitamin D status. The recommendation for vitamin D intake differs between countries in Europe and ranges from 0 to 15 µg for different population groups [28]. According to a recent report of the Institute of Medicine, the RDA for vitamin D should be 15 µg/day for children greater than 1 year old and adults, and 20 µg/day for the elderly [29]. As such, there is a renewed interest in improving dietary intake in an effort to meet these recommendations. Additionally, if we are to counteract population level vitamin D deficiency enhancement of dietary intake is the only feasible mode.

The main objective of the present review is to provide an overview of the vitamin D containing foods available, to include natural, fortified and enriched sources. It will describe the randomized controlled trials that have been performed with vitamin D enhanced foods and the effectiveness of these in raising serum 25(OH)D levels. The current regulations governing the addition of vitamin D to foods in Europe will also be outlined. 

## 2. Food Intakes and Sources of Vitamin D

A recent trans-European examination of nutrient intake patterns showed dietary intakes of vitamin D to differ markedly between countries. Intakes were significantly higher in Nordic countries including Sweden, Denmark and Norway than in Mediterranean regions such as Italy and France [30]. Intakes of less than 3.0 µg/day and 2.0 µg/day have been reported in Italian and Spanish adults [31,32]. Older men in Finland have some of the highest dietary intakes in Europe at 9.0 µg/day [33]. Recent estimates from Ireland revealed intakes ranging from 3.9 µg to 8.5 µg/day in adults, with an increase observed with age [34]. In the US, mean dietary consumption for adults ranges from 3.6 to 5.6 µg/day [35], lower than the population mean of 5.8 µg/day in Canada [36]. Intakes in Japanese adults appear higher, at >6 µg of vitamin D per 1000 kilocalories of energy intake per day [37]. 

Foods that make the highest contribution to dietary intakes of vitamin D vary from country to country according to habitual dietary patterns. In Norway, the predominant food sources are fish and fats [38], as is the case in Finland where liquid milk and dairy products also contribute significantly [33]. In the UK, oily fish (24%), followed by meat and meat products (22%) and cereal and cereal products (21%) contribute the highest percentage to average daily vitamin D intakes in adults [39]. For Irish adults, meat (30%), fish (12%) and spreads (10%) made the most significant contributions of all food groups [34]. In Spain the main source is fish which accounts for 65% of intake [31]. This can be contrasted with the US where vitamin D fortified milk makes by far the highest contribution to intakes (58% in men, 39% in women) [40], figures comparable to those of Canada where milk products account for 49% of dietary vitamin D intake [36]. In Japan, the major sources of dietary vitamin D intake are fish/shellfish (79%) and eggs (9%) [41], though fish consumption has been shown to contribute >90% of vitamin D intake in an elderly Japanese cohort [42]. 

People are often reliant on dietary intake of vitamin D to satisfy their requirements, however foods naturally rich in vitamin D are few in number and in many cases not widely consumed. Table 1 illustrates the levels of vitamin D generally found in a number of food sources as reported in food consumption databases and in the scientific literature. As can be seen in Table 1, the richest sources include fish liver oils, oily fish, egg yolk, and wild mushrooms. However, even in those foods considered the richest sources of vitamin D, the levels are highly variable. For example, the content of vitamin D in fish can vary significantly both between and within species and according to whether they are wild or farmed; the vitamin D content of farmed salmon has been shown to be only approximately 25% that of wild salmon [43]. 

**Table 1:** Vitamin D content of selected foods.

Food	Vitamin D Content (µg/100 g)
Fish	
Cod liver oil	210.0 [44]-250.0 [45]
Salmon, wild (Pacific)	13.1 [45]-24.7 [43]
Salmon, wild (Atlantic)	5.9 [44]
Salmon, farmed *	6.0 [43]
Herring, Baltic, raw	5.7-15.4 [46]
Kipper fillets, raw	8.0 [44]
Mackerel, raw	8.8 [44]-16.1 [45]
Sardines, canned in brine	4.6 [44]
Tuna, canned in oil	6.7 [45]
Anchovy, canned in oil	1.7 [45]-3.0 [44]
Cod	Trace [44]-2.6 [43]
Sole	Trace [44]-2.8 [45]
**Mushrooms ^†^**	
Mushrooms, wild edible (*Cantharellus tubaeformis*)	13.6 [46]-29.8 [47]
Mushrooms, chanterelle	5.3 [45]-14.2 [48]
Mushrooms, white button (*Agaricus bisporus*)	0.2 [45]
Mushrooms, white button (*Agaricus bisporus*), UV irradiated	11.9 [49]
**Dairy**	
Milk, whole, unfortified	0.1 [45]
Milk, fortified	1.3 [45]-2.0 [50]
Cheese, cheddar	0.3 [44]-0.6 [45]
Yoghurt, plain	0.1 [45]
**Meat and Eggs**	
Liver, beef, raw	1.2 [45]
Beef, rib eye steak, raw	0.1 [45]
Pork, cured bacon, raw	1.6 [45]
Ham	0.7 [45]-1.1 [44]
Chicken, breast, raw	0.1 [45]
Turkey, slices	2.2 [44]
Butter	1.5 [45]
Egg, whole, raw	1.8 [44]-2.05 [45]
Egg, yolk, raw	4.9 [44]-5.4 [45]
**Cereal products**	
Breakfast cereals, fortified	2.8-8.3 [44]
Bread, fortified ^†^	3.0 [51]

* Levels dependent on feed; ^†^ Vitamin D in the form of vitamin D_2_. Note: Vitamin D intakes are discussed in terms of micrograms (μg) or International Units (IU). In Europe μg are most commonly adopted as the measure of vitamin D. In order to convert μg to IU multiply by 40.

Fortified foods are those to which one or more essential nutrients have been added, whether or not it is normally contained in the food, for the purpose of preventing or correcting a demonstrated deficiency [52]. Enriched foods are those that have nutrients that were originally lost during processing restored and are sometimes considered interchangeably with fortified foods [53]. It is also possible to enhance animal products by feeding animals vitamin D supplemented feed, thereby increasing the levels of vitamin D present in the edible products. In the current review, unless otherwise stated, we will consider animal and non-animal fortified foods, and enriched foods under the umbrella term of vitamin D-enhanced foods. 

At present, the number and variety of vitamin D fortified foods available on the market differs significantly between countries but most commonly includes milk, breakfast cereals and margarines. The relative deficit of vitamin D fortified foods can be partly attributed to the country-specific policies on food fortification which are not yet unified. Most individual countries have their own national policies, even within Europe, which tend to limit the number or type of foods that can be fortified, as will be discussed later in the review. Even in those countries where vitamin D food fortification is mandatory, current levels of fortification in permitted foods are likely inadequate to satisfy physiological requirements [54]. Furthermore, the small number of foods available is likely to have limited impact on population dietary intake. For example, certain population groups are low consumers of milk, one of the more commonly fortified foods [40]. As such, whilst it is important that currently fortified foods continue to be widely available and consumed, novel methods for producing a wider variety of vitamin D-rich foods are also increasingly sought. 

In recent years, evidence has emerged demonstrating viable new methods for enhancement of foods with vitamin D. Fungi can produce considerable vitamin D_2_ levels when exposed to UVB light through the conversion of natural ergosterol, the rate depending on the irradiation dose and temperature [55,56]. The vitamin D content in these irradiated mushrooms appears relatively stable when refrigerated [57] and the retention of vitamin D in mushrooms following cooking and storage has been reported as high, at 86% and greater [46]. Even after 6 years of storage, dried mushrooms appear to retain much of their vitamin D content [48]. Success in terms of vitamin D enhancement of animal products has been seen with pigs, fish and hens. Increasing the dietary vitamin D_3_ content of pigs diets can produce meat and liver with higher levels of vitamin D_3_[58,59]. Similarly, the levels of vitamin D in fish can be improved through the feeding of vitamin D_3_-rich feed, with >10% of the dietary content of vitamin D_3_ retrieved in the whole fish [60]. The vitamin D content of eggs can also be safely increased by feeding hens vitamin D_3_-rich diets. The consumption of one of these eggs could provide up to 2.8 μg of vitamin D, approximately 3 times that of a typical egg, with scope for increasing this even further [61,62]. The preservation of vitamin D_3_ and 25-hydroxyvitamin D_3_ in egg yolk after cooking is high and only modestly affected by storage, with losses of <10% in both cases [46]. Little work has been done on modelling the impact that the widespread introduction of such enhanced foods might have on the vitamin D intakes of populations. To assess the potential effectiveness of such eggs in increasing vitamin D intakes, we simulated the increase that would result from the consumption of enhanced eggs by Irish adults. Based upon current estimated intakes of 112 g of eggs and egg dishes per week, the consumption of eggs with a vitamin D content of 2.8 µg *versus* 1.1 µg (per 60 g egg) would result in a weekly population intake of 5.2 µg vitamin D from egg products *versus* the current estimated intake of 2.1 µg. By excluding those people that do not consume egg products, intake increases from 4.2 µg to 10.8 µg per week. Further assessment of the potential impact of a range of vitamin D enhanced foods on population intakes of this vitamin is certainly warranted. To this end we have performed food modelling work which will be discussed in the section examining the regulation surrounding the sale of foods enhanced with vitamin D.

## 3. Foods Enhanced with Vitamin D

As outlined above, it is feasible to enhance a variety of foods with vitamin D; however, the biological significance of consumption of these foods needs to be assessed. In order to assess the efficacy of vitamin D enhanced products in increasing serum 25(OH)D concentration, a number of randomized controlled trials (RCTs) have been conducted in recent years. The present review focuses on the RCTs which provided at least 10µg of vitamin D_2_ or D_3_ each day with foods that were enhanced with vitamin D [63,64,65,66,67,68,69,70,71] (Table 2). A literature search was conducted using the PubMed and Medline databases up to May 2011 for studies in the English language. Keywords used included “vitamin D” and foods that could be fortified with vitamin D, such as “cheese”, “bread”, “orange juice”, “yogurt”, “spreads” and “margarine”, “cereals”, and “mushrooms”.

In general, the majority of the studies concentrated on markers of bone metabolism as well as vitamin D status and as a consequence most analysed serum 25(OH)D, PTH and calcium concentration; some included collection of a three day food diary. Most of the interventions were three to twelve weeks duration and were performed during winter months, when the cutaneous synthesis of vitamin D is low and does not contribute to circulating levels of 25(OH)D. Two out of nine studies had a longer duration of twenty-four months. Across the nine studies volunteers were generally healthy, representing both genders and age groups ranging from 18 to 87 years (Table 2).

**Table 2:** Change in serum 25(OH)D after intake of foods enhanced with vitamin D.

Food source	References	Daily dose (µg) vitamin D (µg vitamin D/100 g product)	Duration and population	Study groups (product portion)	Change from baseline of serum 25(OH)D (%)
Fortified milk	Chee *et al.*, 2003 [64]	10 µg	24 months	1. vitamin D_3_ + Ca-fortified skimmed milk	25.0 ^#^
		(2.2 µg/100 g)	50-65 years, postmenopausal females	2. Usual diet	4.1 ^#^
			*n* = 200		
	Daly *et al.*, 2006 [65]	20 µg	24 months	1. Vitamin D_3_ + Ca-fortified UHT milk, reduced fat	7.4 ^#^
		(5.0 µg/100 g)	50-87 years, ambulatory community-living (male)	2. Usual diet	−19.9 ^#^
			*n* = 167		
Fortified yogurt drink	Nikooyeh *et al.*, 2011 [68]	25 µg	12 weeks, October-March	1. Vitamin D_3_-fortified yogurt drink	75
		(5.0 µg/100 g)	30-60 years, diabetic subjects (fasting blood glucose ≥126 mg/dL)	2. Vitamin D_3_ + Ca-fortified yogurt drink	67.6
			*n* = 90	3. Plain yogurt drink	−10.6
Fortified cheese	Johnson *et al.*, 2005 [66] (study 1)	15 µg	2 winter months	1. Vitamin D_3_-fortified process cheese	−8.7
		(17.6 µg/100 g)	≥60 years, subjects (total serum cholesterol <240 mg/dL)	2. Placebo process cheese	10
			*n* = 110	3. No cheese	5.6
	Wagner *et al.*, 2008 [71]	100 µg *	8 weeks, January-April	1.Vitamin D_3_-fortified regular fat cheddar cheese ***	128.8
		(2083.3 or 1690.8 µg/100 g) **	18-60 years, healthy subjects	2. Vitamin D_3_-fortified reduced fat cheddar cheese	120.7
			*n* = 80	3. Vitamin D_3_ supplement to be taken with food	106.5
				4. Vitamin D_3_ supplement to be taken without food	111
				5. Placebo regular fat cheddar cheese	−7.8 ^##^
				6. Placebo supplement	
Fortified orange juice	Tangpricha *et al.*, 2003 [69] (study 2)	25 µg	12 weeks, commenced in March	1. Vitamin D_3_ + Ca-fortified orange juice	150
		(10.4 µg/100 g)	22-60 years, healthy subjects	2. Placebo Ca-fortified orange juice	45
			*n* = 30		
	Biancuzzo *et al.*, 2010 [63]	25 µg	11 weeks, commenced in February	1. Vitamin D_3_ orange juice ^ + placebo capsule	71.5
		(10.6 µg/100 g)	18-79 years, healthy subjects	2. Vitamin D_2_ orange juice + placebo capsule	67.1
			*n* = 105	3. Vitamin D_3_ capsules + placebo orange juice	42.9
				4. Vitamin D_2_ capsules + placebo orange juice	65.1
				5. Placebo capsule + placebo orange juice	−8.6
UV enhanced mushrooms	Urbain *et al.*, 2011 [70]	100 µg *	3 weeks + 2 weeks follow up, January-March	1. Vitamin D_2_ soup + placebo orange juice ***	50.0 ^#^
		(191.0 µg/100 g) **	Healthy female and male <45 years	2. Placebo soup + vitamin D_2_ supplement in orange juice	76.7 ^#^
			*n* = 27	3. Placebo soup + placebo orange juice	−28.9 ^#^
Fortified bread	Natri *et al.*, 2006 [67]	10 µg	3 weeks, February-March	1. Vitamin D_3_-fortified wheat bread (85 g)	65.0 ^###^
		(27.3 µg/100 g)	25-45 years, healthy females (25(OH)D < 58.1 nmol/L)	2. Vitamin D_3_-fortified rye bread	59.0 ^###^
			*n* = 41	3. Regular wheat bread + vitamin D_3_ supplement	78.0 ^###^
				4. Regular wheat bread	−1.2 ^###^

* equivalent to a daily dose; ** ingested in one weekly dose; *** all portions served once a week; ^#^ measurement at the end of the intervention; ^##^ combined placebo groups; ^###^ estimated baseline 25(OH)D; ^ all orange juice in this study contained 350 mg Ca/236.6 mL.

In the current literature the most commonly used vehicles for vitamin D fortification are dairy products (milk, cheese and yogurt). Two long term studies (24 months) used milk fortified with vitamin D_3_ to deliver 10 µg or greater per day [64,65]. In both studies, 25(OH)D concentrations increased for the supplemented group compared to baseline; however, the relative increases varied between the studies (25.0% *vs.* 7.4%) which may be as a result of very different study populations. It should be noted that in the study by Daly and colleagues a 20% decrease in serum 25(OH)D was reported for the control group. In general, compliance was good, with only a small number of dropouts reported despite the necessity to consume a large volume of milk (400 mL/day). Both studies concluded that vitamin D was bioavailable and improved 25(OH)D status and markers of bone turnover. Similarly, daily intake of 25 µg of vitamin D_3_ in a yogurt drink with or without added calcium significantly increased serum 25(OH)D_3_ concentrations after 12 weeks by 75.0% and 67.6%, respectively, compared to a decrease of 10.6% in the control group. In addition, the glycaemic status in diabetic patients was improved [68]. Acceptance of the drink was high among volunteers and no adverse effects were observed. 

Fortification of cheese with vitamin D has been examined in two separate eight-week studies showing diverse results. In the study by Johnson and colleagues, consumption of fortified cheese (15 µg vitamin D) resulted in an 8.7% decrease in their serum 25(OH)D [66]. This was an unexpected finding that the authors attribute to a higher baseline value of 25(OH)D in the supplemented group. Additionally no changes in serum PTH were found. The second study by Wagner *et al.* showed an approximate 120% increase in serum 25(OH)D concentration after a weekly dose of 700 µg vitamin D fortified cheese [71]. In this study serum PTH decreased and there were no changes in serum calcium concentration; additionally no dropouts or adverse side effects observed were reported. However, in the study by Johnson and colleagues there were five dropouts in the cheese group: the reasons for withdrawal included gastrointestinal problems, a dislike for the saltiness of the cheese and medical advice [66]. Indeed, as cheese typically contains relatively high levels of salt and saturated fat, a portion size closer to that usually recommended for cheese (30 g) rather than what was used by Johnson *et al*. (85 g) may have been more appropriate [66]. Overall, cheese could be a feasible food for the improvement of vitamin D status, but a more realistic amount and vitamin D dose should be used in future studies. To summarise, vitamin D was bioavailable from all studied dairy products; enhanced milk and yogurt or a very high weekly intake of enhanced cheese improved vitamin D status, whilst consumption of 15 µg of vitamin D in cheese was insufficient to produce a significant increase in serum 25(OH)D. 

Due to lactose intolerance or low dairy product consumption in some countries, new foods should be considered as possible vehicles for enhancement with vitamin D. Two RCTs using orange juice fortified with 25 µg of vitamin D showed a positive effect on serum 25(OH)D concentration [63,69]. In the study of Tangpricha *et al*., 25(OH)D increased by 150% after 12 weeks supplementation [69]. The second study reported that ingestion of 25 µg vitamin D_2_ and D_3_ fortified orange juice for 11 weeks resulted in a rise in serum 25(OH)D concentration by 67.1% and 71.5%, respectively [63]. Serum PTH decreased only in the first study, while calcium concentration remained stable in both interventions. No adverse effects were reported in either study. The fortification of orange juice with vitamin D seems a promising method of boosting serum 25(OH)D concentration. However, replication of the above studies using a daily dose in line with the RDA for vitamin D is warranted. 

Another non-dairy vitamin D enhanced product that has received attention recently is mushrooms. Vitamin D_2_ from wild grown mushrooms was well absorbed and bioavailable in humans [72]. This study used wild and not enhanced mushrooms, and therefore has not been included in this review that focuses on enhanced vitamin D foods. However, a recent paper has investigated the effect of a weekly dose of 700 µg of vitamin D from vitamin D_2_-enhanced mushrooms in a soup [70] and reported a 50% increase in serum 25(OH)D concentration. There were no changes in serum PTH and calcium concentration and no adverse effects were observed. Additionally, the soup was well tolerated by volunteers. The limitations of this study include the low number of subjects and the high vitamin D dose ingested weekly. Nonetheless, this study demonstrated that vitamin D from mushrooms was bioavailable and shows promise as an alternative to supplements in maintaining serum 25(OH)D concentration.

Bread fortified with vitamin D could also serve as a good source of vitamin D due to its common consumption. Promising results were obtained following daily consumption of 125 µg of vitamin D_3_ enhanced bread for 12 months in older adults [73], however the details of the study are not included in Table 2 as it was a single-arm design. To date, only one RCT has investigated the effect of supplementation of wheat and rye bread with 10 µg of vitamin D_3_ per daily 85 g portion (equivalent to four thin slices) [67]. In this study serum 25(OH)D increased by approximately 60% after 3 weeks in both groups that received fortified bread. No differences in serum PTH were found, while calcium concentration decreased in the fortified rye bread group. Three subjects dropped out of the study due to difficulties with compliance. In conclusion, fortified bread increased serum 25(OH)D concentration, with rye and wheat bread equally effective.

Finally, the aforementioned RCTs show the potential of a number of vitamin D enhanced foods to increase vitamin D status. Overall, fortification was the principal strategy adopted but the potential of UV light enhanced mushrooms to boost vitamin D status was more recently explored. A comparison between the studies is difficult to perform because of variability in the population characteristics (*i.e.*, age, gender), dose of vitamin D provided, intervention period and methods used for 25(OH)D determination [74,75]. The majority of the studies provided a daily supply of the foods whilst two used a weekly dose. However, the impact of the frequency of dose has not been studied in relation to any of the above foods. With respect to intake of vitamin D supplements it has been demonstrated that the frequency of intake has an impact on 25(OH)D serum concentrations [76], illustrating the imperative need to study this in the vitamin D enhanced foods. Overall, in the enhanced food a higher dose was more effective in increasing 25(OH)D concentration; however, for short term studies (3 weeks) the increase seemed to be independent of dose. The majority of the foods in the selected RCTs were well tolerated by the study subjects. After the ingestion of different vitamin D fortified/enriched foods, PTH decreased or did not change, while serum calcium concentration remained stable or in the case of the rye bread group decreased, highlighting no safety risks with the use of these vitamin D enhanced products. Milk, yogurt, orange juice, mushrooms and bread proved to be good matrices for supplementation with vitamin D at the level of 10 µg (milk, bread), 25 µg (orange juice and yogurt) or or 100 µg (mushrooms). More studies are needed to assess the effectiveness of vitamin D fortified cheese, with the use of a more feasible serving size. Finally, more RCTs, using these foods, should be conducted to investigate the link between vitamin D and other disease markers beyond those related to bone health and vitamin D status.

## 4. Vitamin D_2_ and D_3_ Bioavailability

Until relatively recently it was widely believed, based upon anti-rachitic findings, that vitamin D_2_ and D_3_ were equipotent and could be considered interchangeably. However, evidence is accumulating to suggest the two forms may vary in their bioefficacy and the importance of this must be considered when discussing potential food matrices. A number of recent studies addressing the potency of the two calciferols have found D_3_ to be more effective in raising serum 25(OH)D levels. In a group of vitamin D insufficient hip fracture patients, a daily capsule of 25 µg D_3_ was more effective than an equal dose of D_2_ in increasing total serum 25(OH)D [77]. Also in older populations, 15 µg daily or 1250 µg monthly of capsular vitamin D_3_ increased serum 25(OH)D more efficiently than an equal dose of D_2_ [78], as has also been shown with a single 7500 µg dose of oral or intramuscular D_3_[79]. The increased ability of a weekly capsule of 1250 µg of D_3_ for 12 weeks to maintain serum 25(OH)D when compared with equimolar D_2_ has also been shown in healthy adults [80]. These findings support those of a number of earlier studies also addressing this topic [81,82]. 

The above findings were not replicated in a number of recent studies in which D_2_ and D_3_ were found to be equally effective in increasing serum 25(OH)D when administered to healthy adults as capsules [63,83] or in orange juice [63] for 11 weeks, or in a high-dose liquid suspension to vitamin D deficient infants for 6 weeks [84]. Despite this, the general consensus that is beginning to emerge is that vitamin D_3_ is the more potent form. However, it is difficult to directly compare the results of the discussed studies given the many confounding factors between them. Most notable in this regard are the different methods employed to analyze serum 25(OH)D, the differing baseline serum levels, the variance in age groups studied, the doses administered, and the duration of supplementation. Overall, more studies specifically designed to address this question are needed before the results can be translated into a public health message.

## 5. Regulation Surrounding the Sale of Foods Enhanced with Vitamin D

From a regulatory perspective, the vitamin D enhanced foods mentioned in this review have typically fallen into two categories (i) fortified foods and (ii) foods that have used technologies to enhance naturally occurring vitamin D levels. However, prior to sale on a commercial scale, there are a series of steps which potential manufacturers must consider. Globally these can vary depending on the region (e.g., Europe, US) and also between countries (e.g., with respect to fortification policies). This review will now briefly outline some of the regulations that must be considered in the EU. 

For fortified foods, addition of both ergocalciferol and cholecalciferol is permitted but consideration must be given to dietary intake from other sources and safe upper levels of intake before agreeing on the levels of addition [85]. At present the IOM has defined 100 μg/day as an upper level for vitamin D intake [29]. Regarding foods which exploit new technologies to enhance naturally occurring levels (as in the case of vitamin D enhanced mushrooms, which were irradiated by UV light), if they are not currently widely consumed within the EU, they would be considered as a “novel food” and subject to approval as “safe for use” by the competent authority in the applicant country and also in other Member States [86].

Thereafter, both categories of food are subject to general food law labelling. Currently, in the EU vitamins and minerals can be declared on food only if they are present in a “significant” amount, *i.e.*, ≥15% of Recommended Daily Allowance (RDA) [87]. According to current legislation in relation to vitamin D, food containing ≥0.75 µg vitamin D/100 g (100 mL) product can be claimed as a “source” of vitamin D, whereas to be declared as “high” in vitamin D, it has to contain at least twice the value of a “source” of vitamin D, *i.e.*, ≥1.5 µg/100 g (100 mL) or 30% RDA [88,89]. For those manufacturers who wish to make further nutrition and health claims, specific procedures and conditions for use are applicable to each claim and are outlined in the appropriate regulations [88]. In brief, there are three categories of permitted health claims (i) article 13 general function claims; (ii) article 13.5 claims based on newly developed scientific evidence; and (iii) article 14 claims referring to the reduction of a risk factor in the development of a disease and children’s development and health. Currently, approved vitamin D health claims relate to vitamin D and bone and teeth health, absorption and utilisation of calcium and phosphorus, normal function of the immune system, cell division, normal growth and development of bone in children, as well as claims referring to the relationship between calcium and vitamin D and the reduction of bone loss in elderly women [90,91,92,93,94]. However, if a vitamin D-enhanced product wishes to bear any of the above claims, it must conform to the aforementioned conditions for use and to as yet unspecified nutrient profiles [87,88].

As mentioned, we conducted food modelling to simulate the intake of vitamin D from enhanced foods in the Irish adult population. Initially the effect of consumption of orange juice enhanced with 10.5 µg vitamin D per 100 g on mean daily intakes was investigated. Consumption of such orange juice would increase mean intakes of vitamin D from 4.7 µg to 7.9 µg per day. In another simulation designed to mimic mandatory fortification policies, in tandem with the orange juice, milk was enhanced at a conservative level of 1 µg/100 g. In this scenario intakes increased to 9.9 µg/day, however, it also resulted in 1% of the population consuming >50 µg vitamin D, thereby reiterating the need to be cognisant of extreme consumers. On the other hand, any mandatory fortification policies would need to be aware of non-consumers of the particular enhanced food group: to this end it may be prudent to introduce fortification at low levels in a number of foods. 

## 6. Conclusions

Overall, the evidence that enhanced vitamin D foods can improve vitamin D status is convincing. A range of enhanced foods were shown to be effective in increasing circulating 25(OH)D concentrations with no signs of adverse effects. Further research and development in such foods is warranted as they have the potential to provide a means to address the vitamin D deficiency pandemic.
